# Supplementary material for: FCHSD2 controls oncogenic ERK1/2 signaling outcome by regulating endocytic trafficking
Source: PLoS Biol. 2020 Jul 17;18(7):e3000778. doi: 10.1371/journal.pbio.3000778 (PMC7390455; doi:10.1371/journal.pbio.3000778)

Full gel image of western bloting

Related to Fig 1A

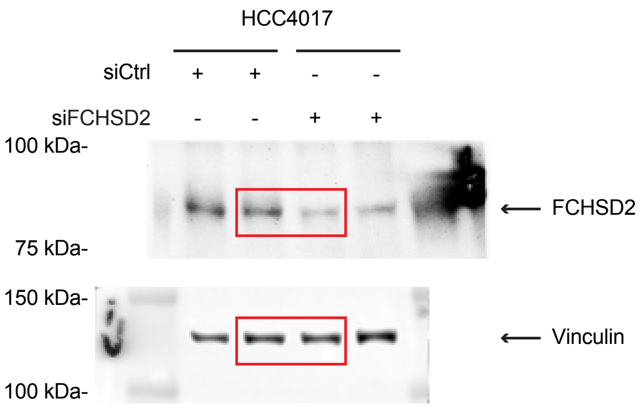

Related to Fig 1F

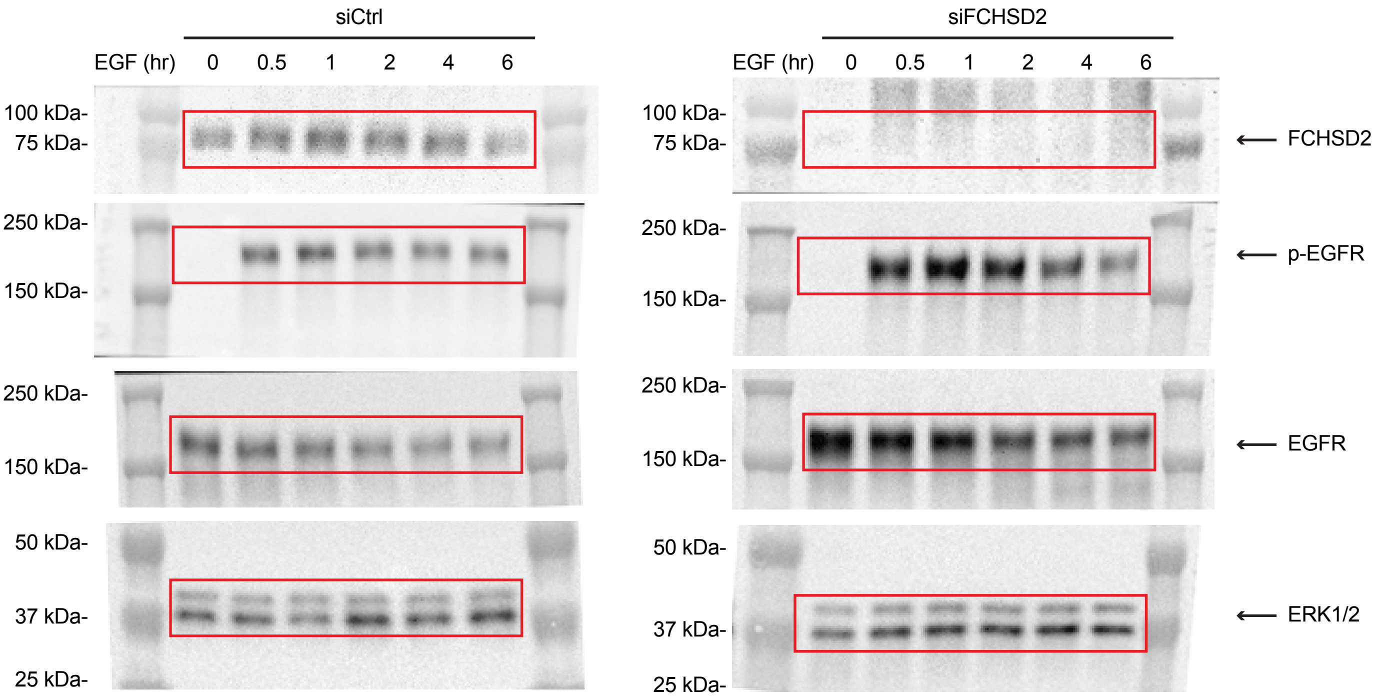

Related to Fig 2B

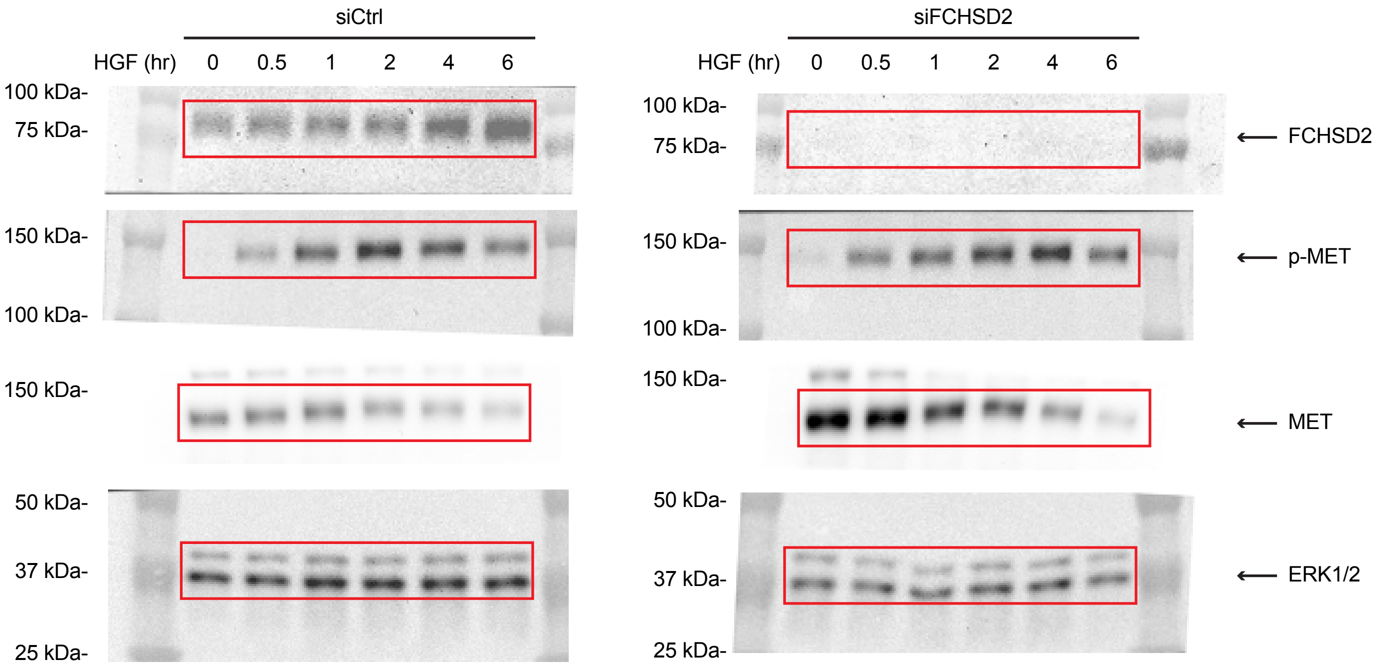

Related to Fig 3B

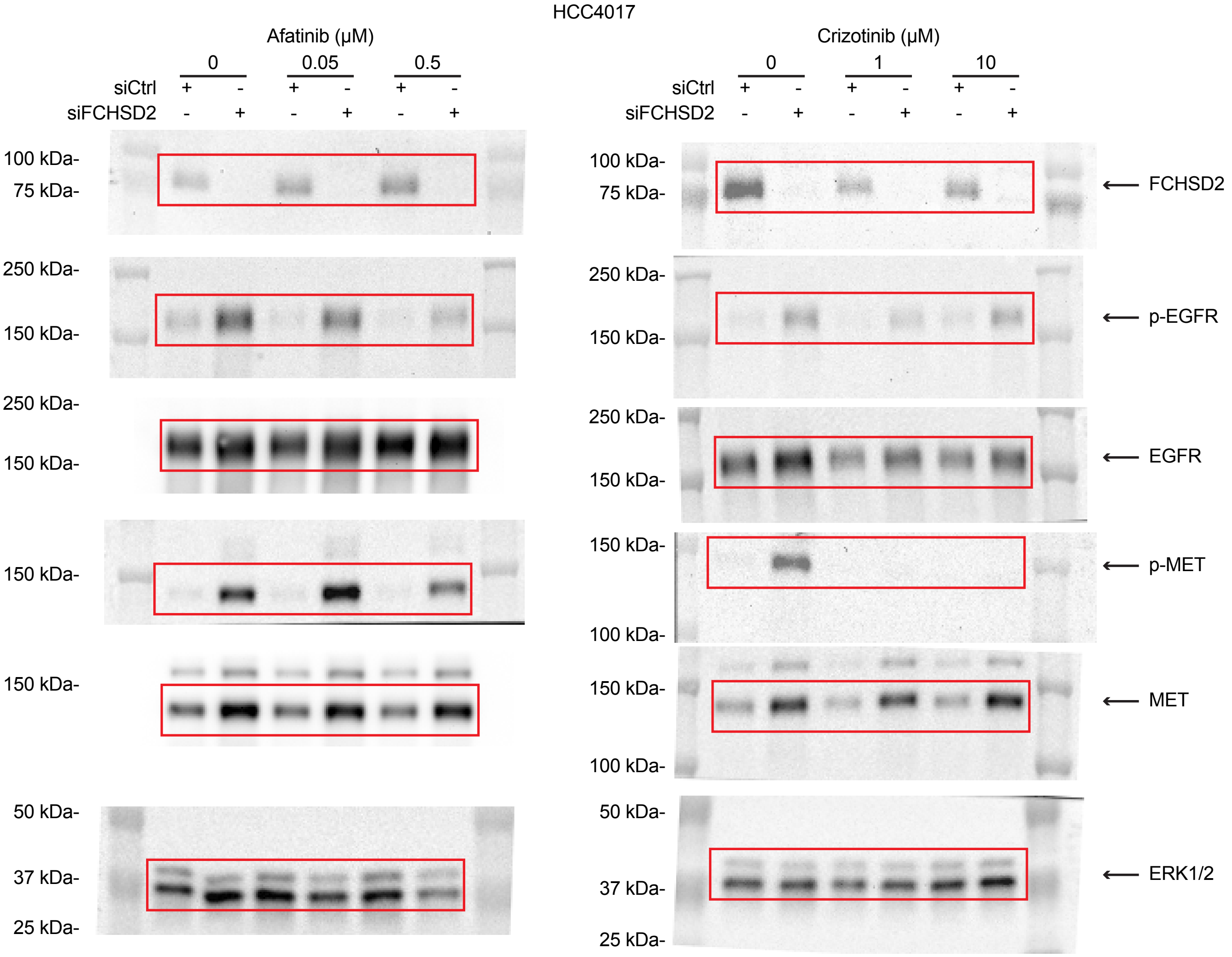

Related to Fig 3D

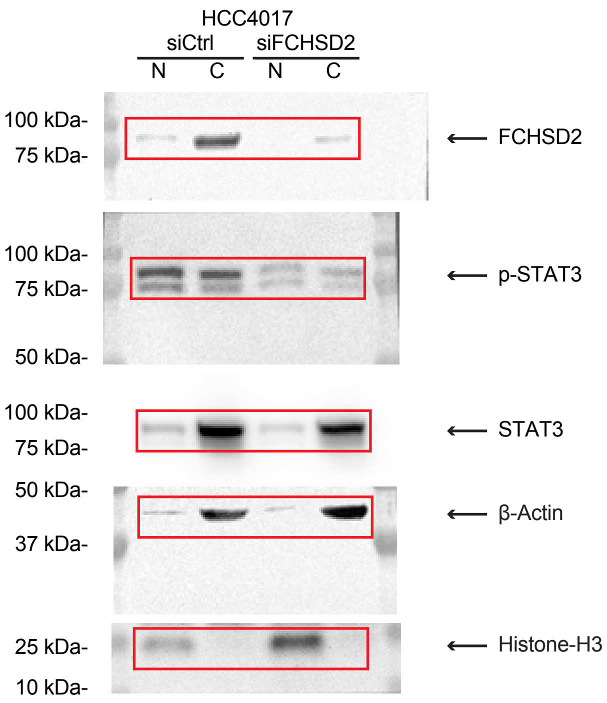

Related to Fig 4A

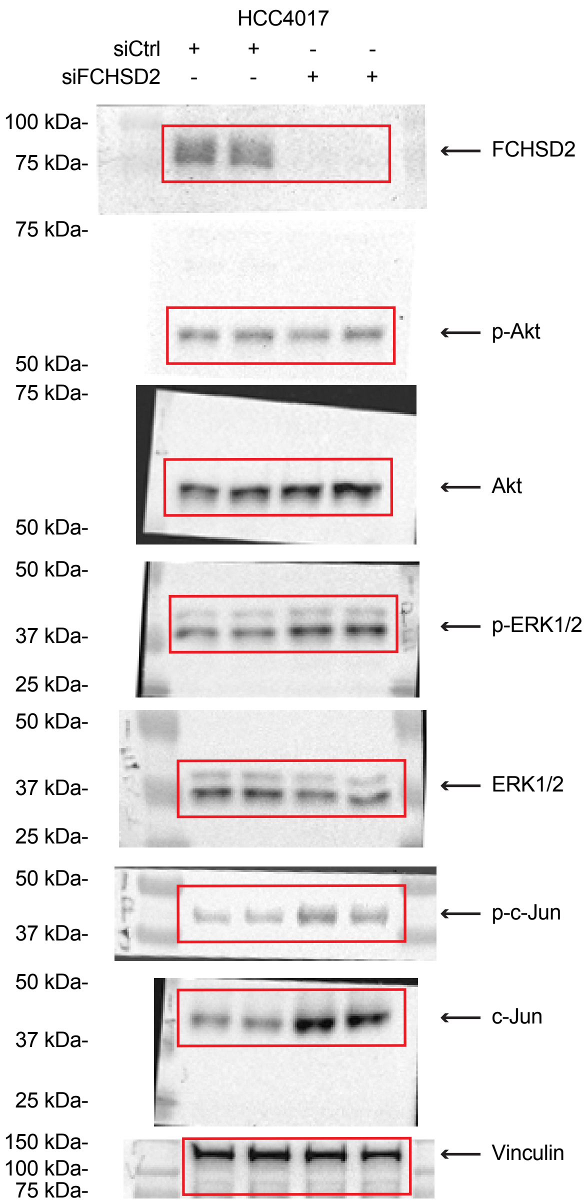

Related to Fig 4D

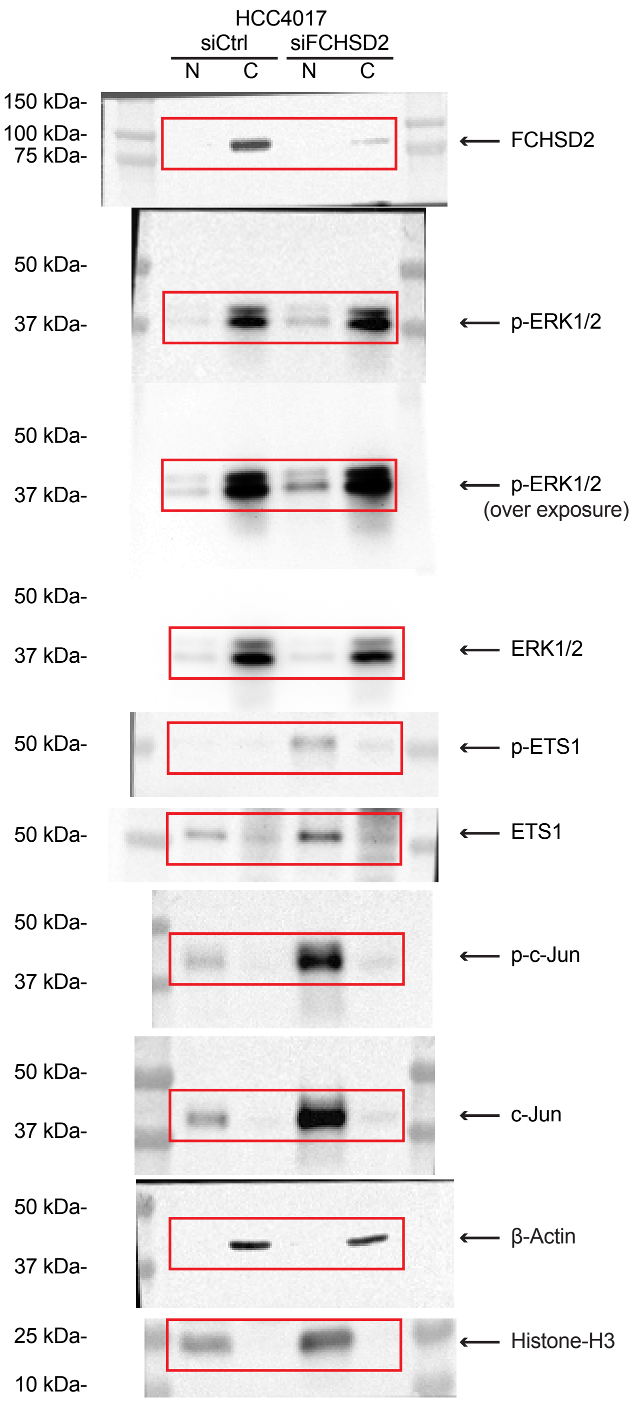

Related to Fig 4F

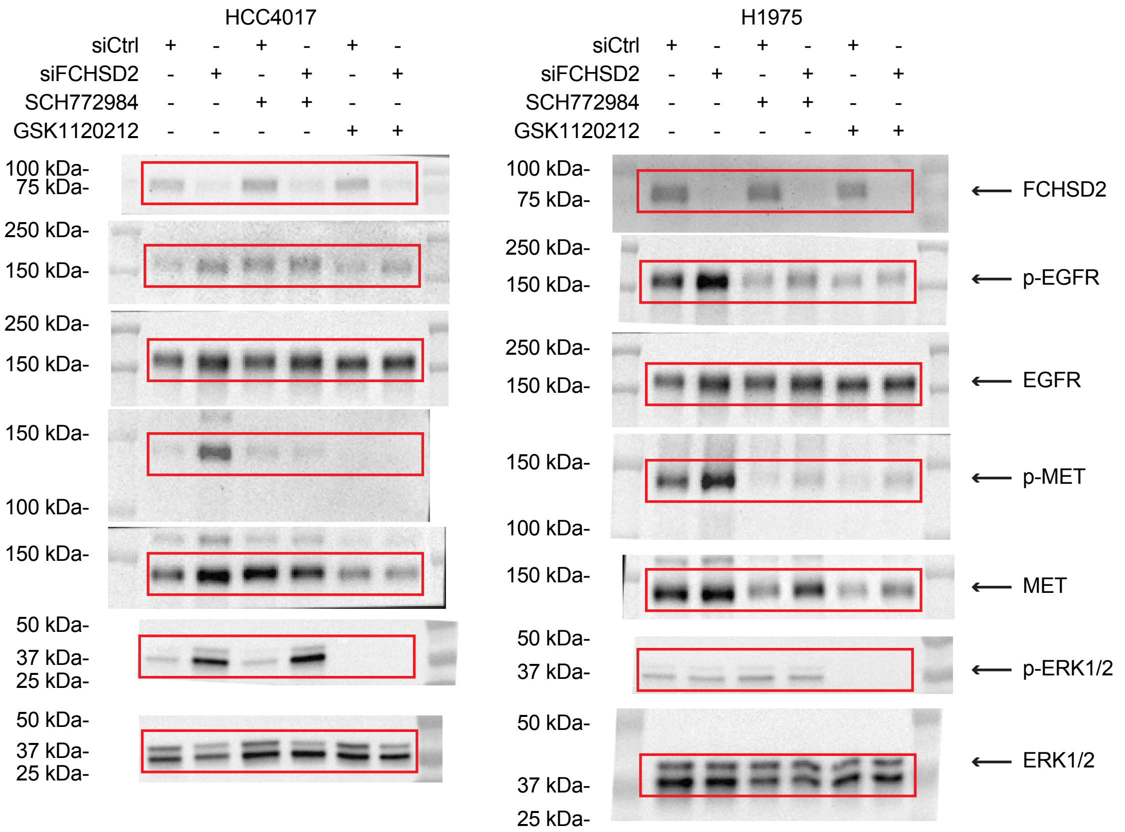

Related to Fig 5B

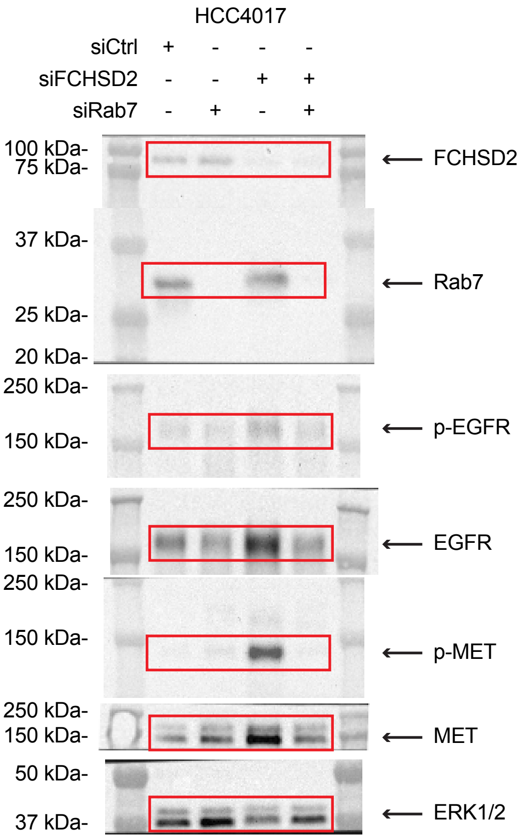

Related to Fig 5C

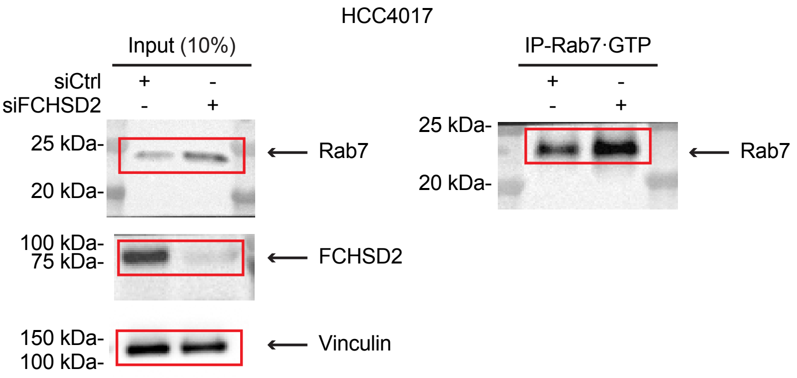

Related to S2A Fig

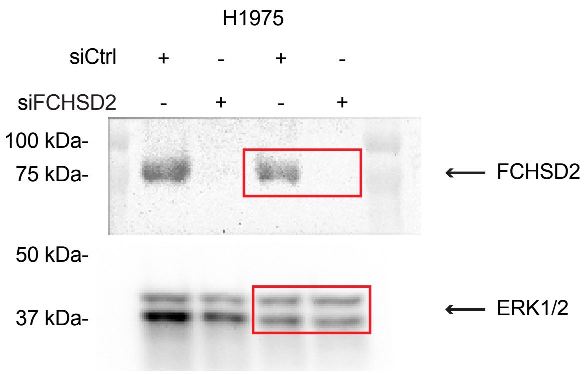

Related to S5 Fig

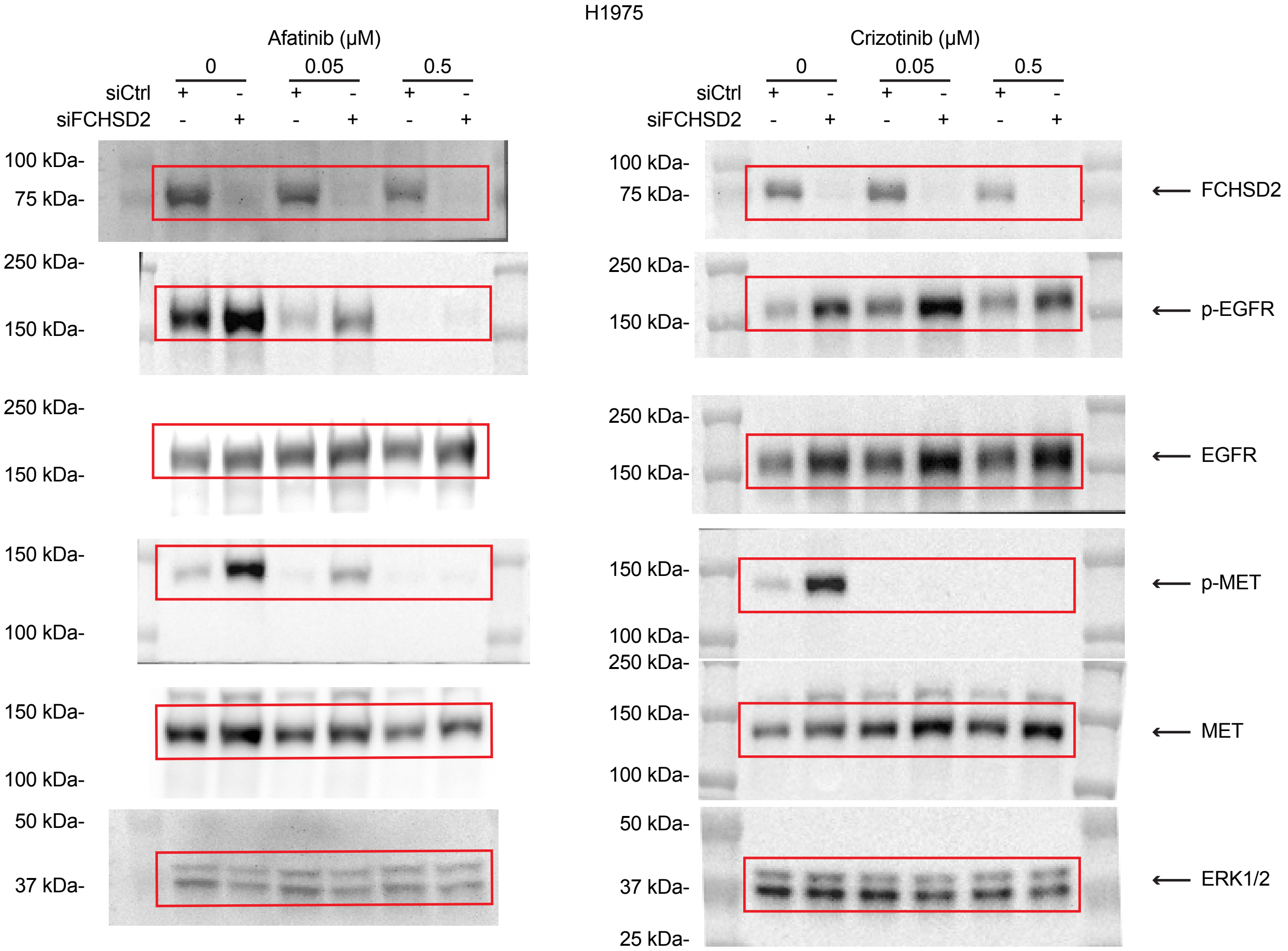

Related to S6 Fig

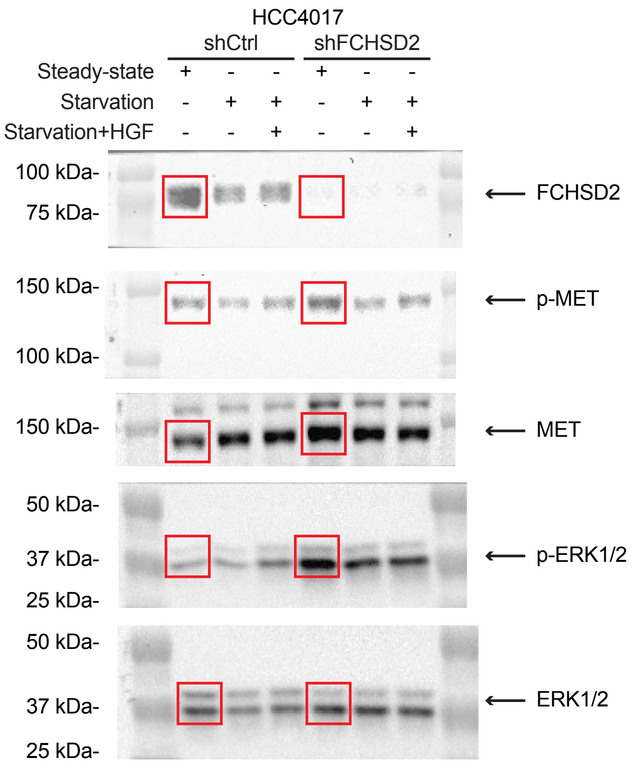

Related to S7 Fig

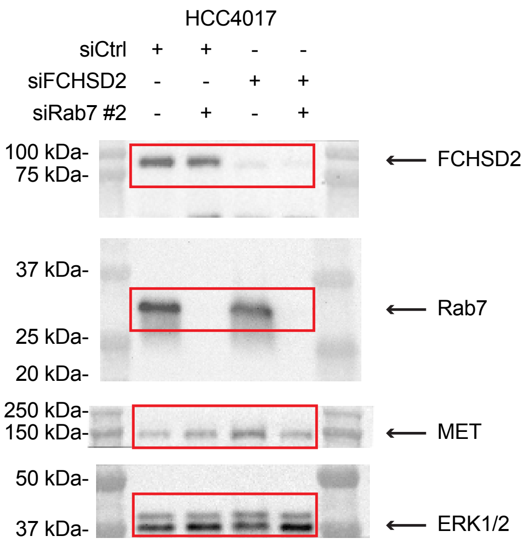

Supplement: S1 Raw Images — The loading order, experimental samples, and molecular weight markers are indicated. The lanes used in the final figure are marked with a red box. (PDF) [file pbio.3000778.s002.pdf]
