# Supplementary figures and images for: FCHSD2 controls oncogenic ERK1/2 signaling outcome by regulating endocytic trafficking
Source: PLoS Biol. 2020 Jul 17;18(7):e3000778. doi: 10.1371/journal.pbio.3000778 (PMC7390455; doi:10.1371/journal.pbio.3000778)

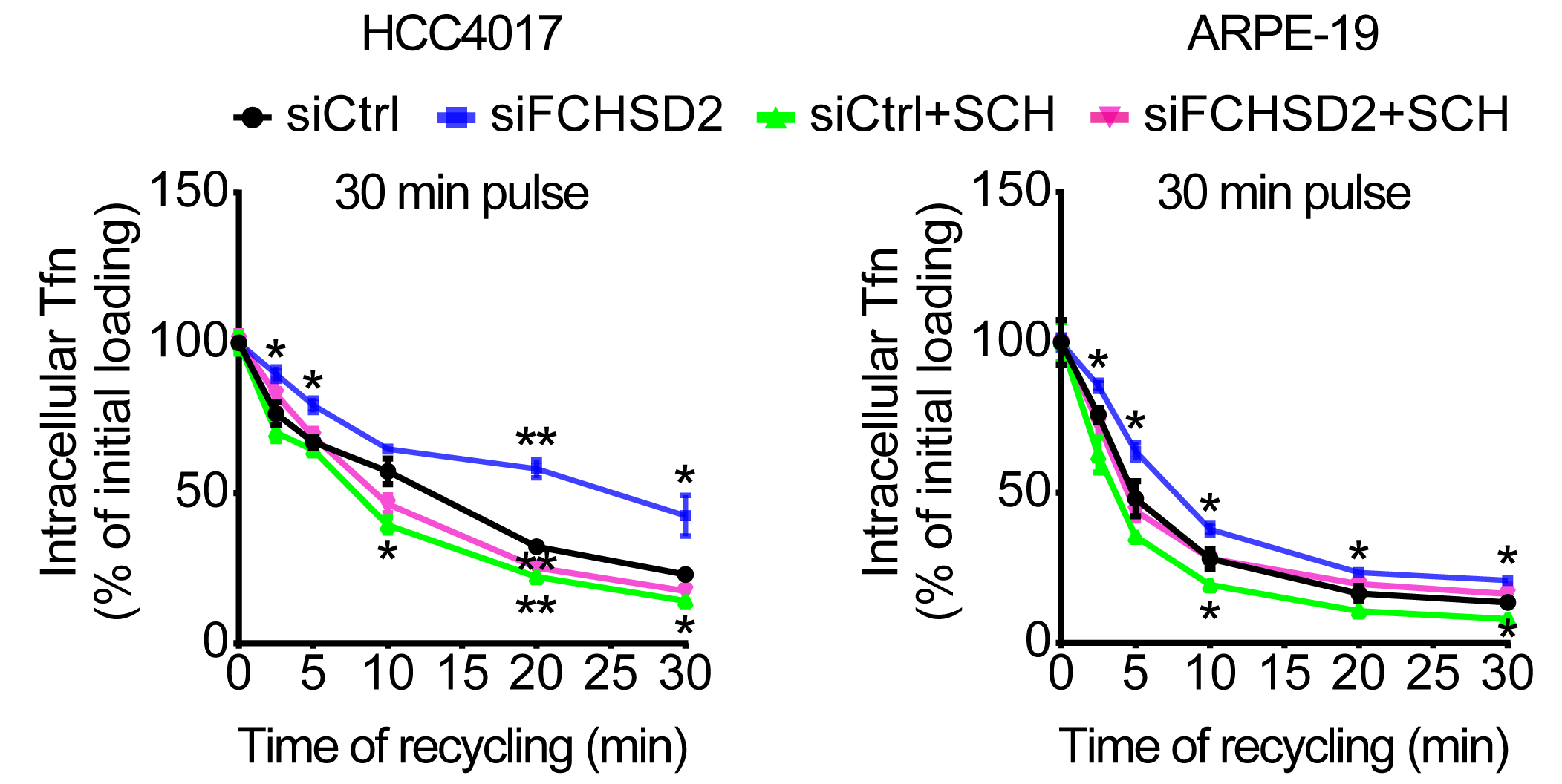

Supplement: S1 Fig — Endocytic recycling of TfnR was measured in control or FCHSD2 siRNA-treated HCC4017 and ARPE-19 cells in the absence or presence of the ERK1/2 inhibitor SCH772984 (10 μM). Cells were pulsed for 30 min with 10 μg/ml biotinylated Tfn, stripped, and reincubated at 37 °C for the indicated times before measuring the remaining intracellular Tfn. Percentage of recycled biotinylated Tfn was calculated relative to the initial loading. Data represent mean ± SEM (n = 3). Two-tailed Student t tests were used to assess statistical significance versus siCtrl. *P < 0.05, **P < 0.005. The underlying data for this figure can be found in S1 Data. ERK1/2, extracellular signal-regulated kinase 1 and 2; FCHSD2, FCH/F-BAR and Double SH3 Domain-Containing Protein; siCtrl, control siRNA; siRNA, small interfering RNA; Tfn, transferrin; TfnR, transferrin receptor. (TIF) [file pbio.3000778.s003.tif]

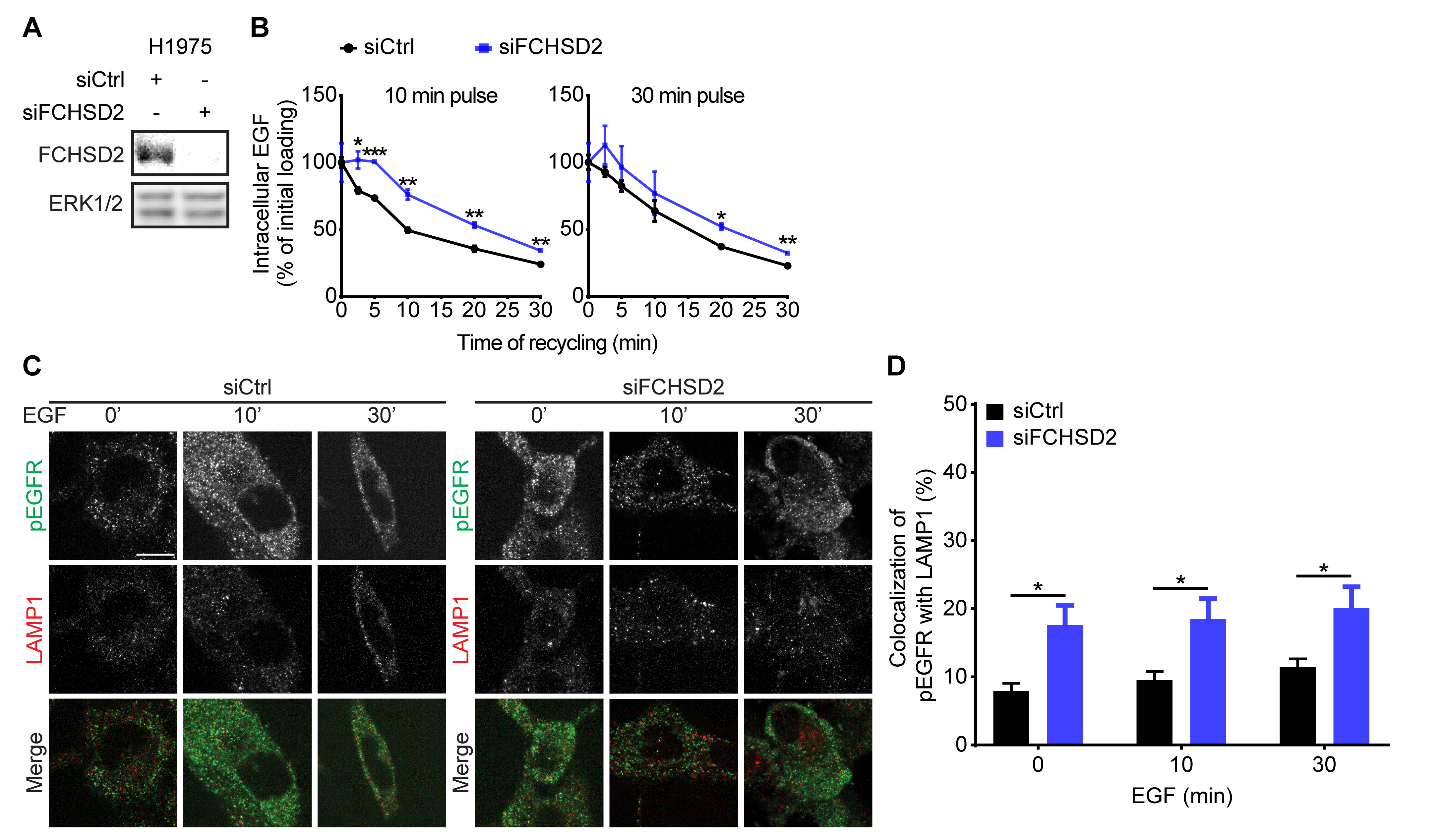

Supplement: S2 Fig — (A) The KD of FCHSD2 in control or FCHSD2 siRNA-treated H1975 cells. (B) Endocytic recycling of EGFR was measured in control or FCHSD2 siRNA-treated H1975 cells. Cells were pulsed for 10 min or 30 min with 20 ng/ml biotinylated EGF, stripped, and re-incubated at 37 °C for the indicated times before measuring the remaining intracellular EGF. Percentage of recycled EGF was calculated relative to the initial loading. Data represent mean ± SEM (n = 3). Two-tailed Student t tests were used to assess statistical significance. *P < 0.05, **P < 0.005, ***P < 0.0005. (C) Representative confocal images of pEGFR and LAMP1 immunofluorescence staining in control or FCHSD2 siRNA-treated H1975 cells. Cells were incubated with 20 ng/ml EGF for 30 min at 4 °C, washed, and re-incubated at 37 °C for the indicated times. Scale bar, 12.5 μm. (D) Colocalization of pEGFR and LAMP1 immunofluorescence staining in the cells as described in (C). Data were obtained from at least 40 cells in total/condition and represent mean ± SEM. Two-tailed Student t tests were used to assess statistical significance. *P < 0.05. The underlying data for this figure can be found in S1 Data. EGF, epidermal growth factor; EGFR, epidermal growth factor receptor; FCHSD2, FCH/F-BAR and Double SH3 Domain-Containing Protein; KD, knockdown; LAMP1, lysosome-associated membrane glycoprotein 1; siCtrl, control siRNA; siRNA, small interfering RNA. (TIF) [file pbio.3000778.s004.tif]

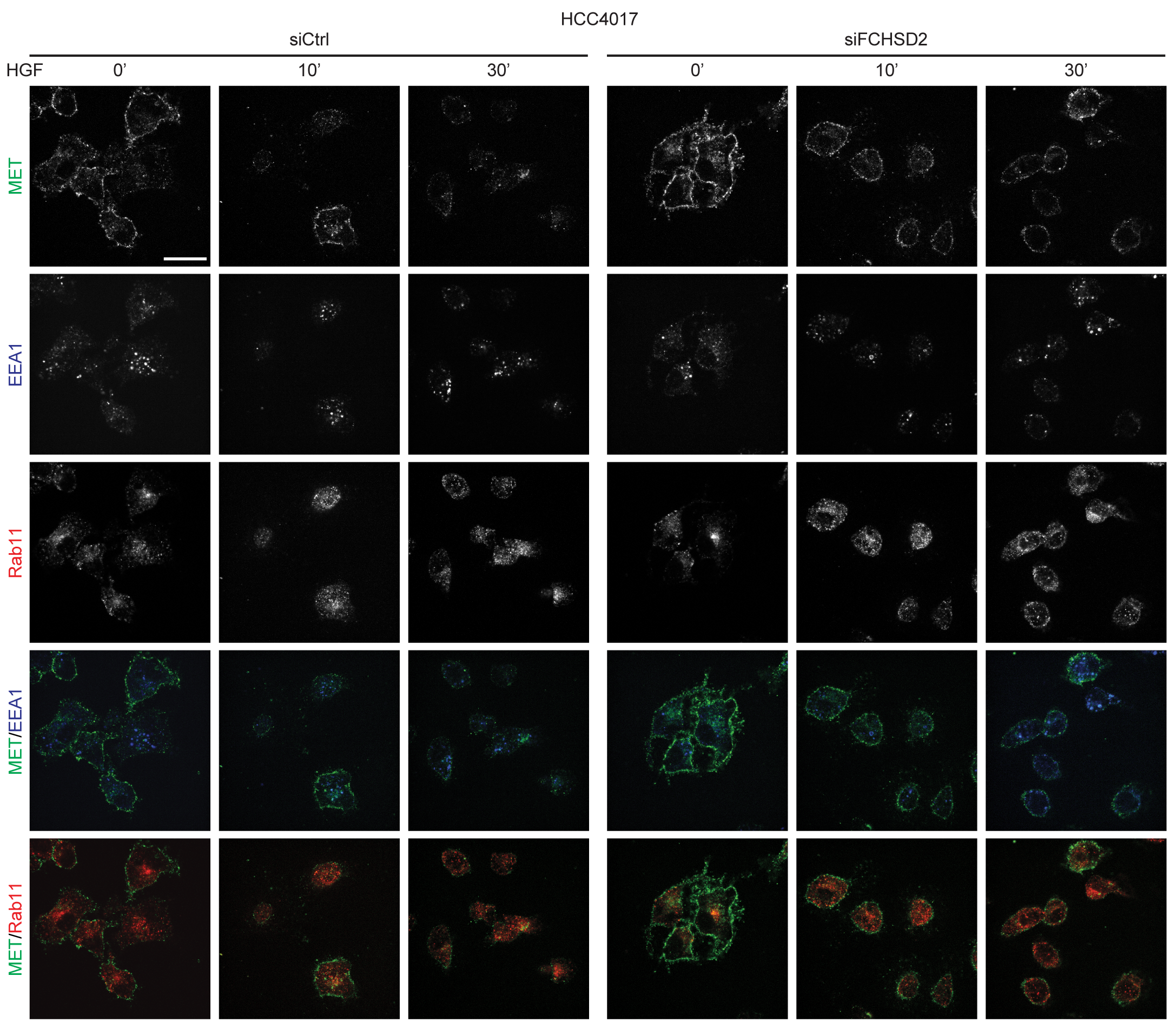

Supplement: S3 Fig — Cells were incubated with 1 μg/ml HGF for 30 min at 4 °C, washed, and re-incubated at 37 °C for the indicated times. Scale bar, 25 μm. Quantified results are shown in Fig 2A. EEA1, early endosome antigen 1; FCHSD2, FCH/F-BAR and Double SH3 Domain-Containing Protein; HGF, hepatocyte growth factor; MET, proto-oncogene c-Met; Rab11, Ras-related protein Rab-11A; siCtrl, control siRNA; siRNA, small interfering RNA. (TIF) [file pbio.3000778.s005.tif]

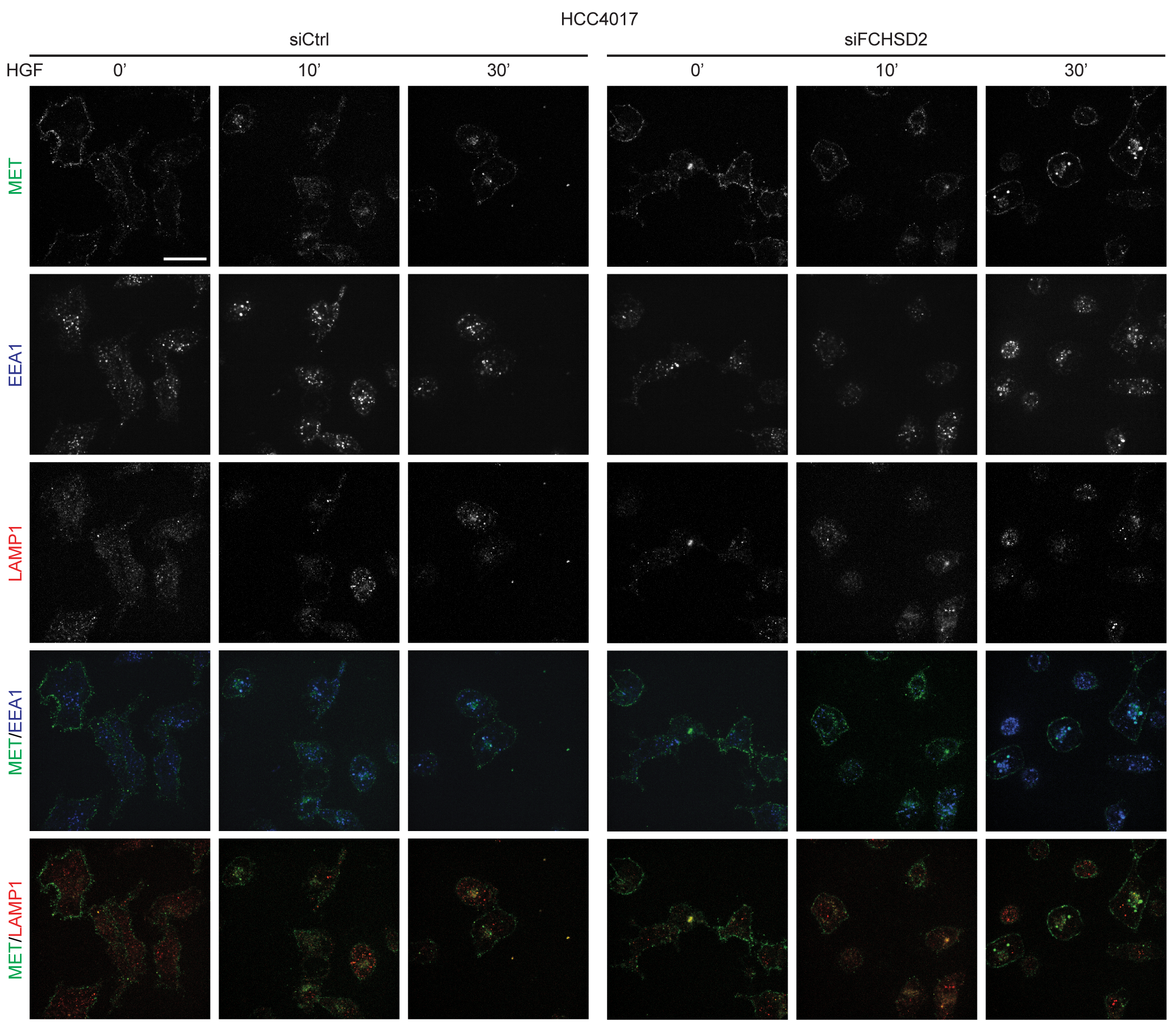

Supplement: S4 Fig — Cells were incubated with 1 μg/ml HGF for 30 min at 4 °C, washed, and re-incubated at 37 °C for the indicated times. Scale bar, 25 μm. Quantified results are shown in Fig 2A. EEA1, early endosome antigen 1; FCHSD2, FCH/F-BAR and Double SH3 Domain-Containing Protein; LAMP1, lysosome-associated membrane glycoprotein 1; MET, proto-oncogene c-Met; siCtrl, control siRNA; siRNA, small interfering RNA. (TIF) [file pbio.3000778.s006.tif]

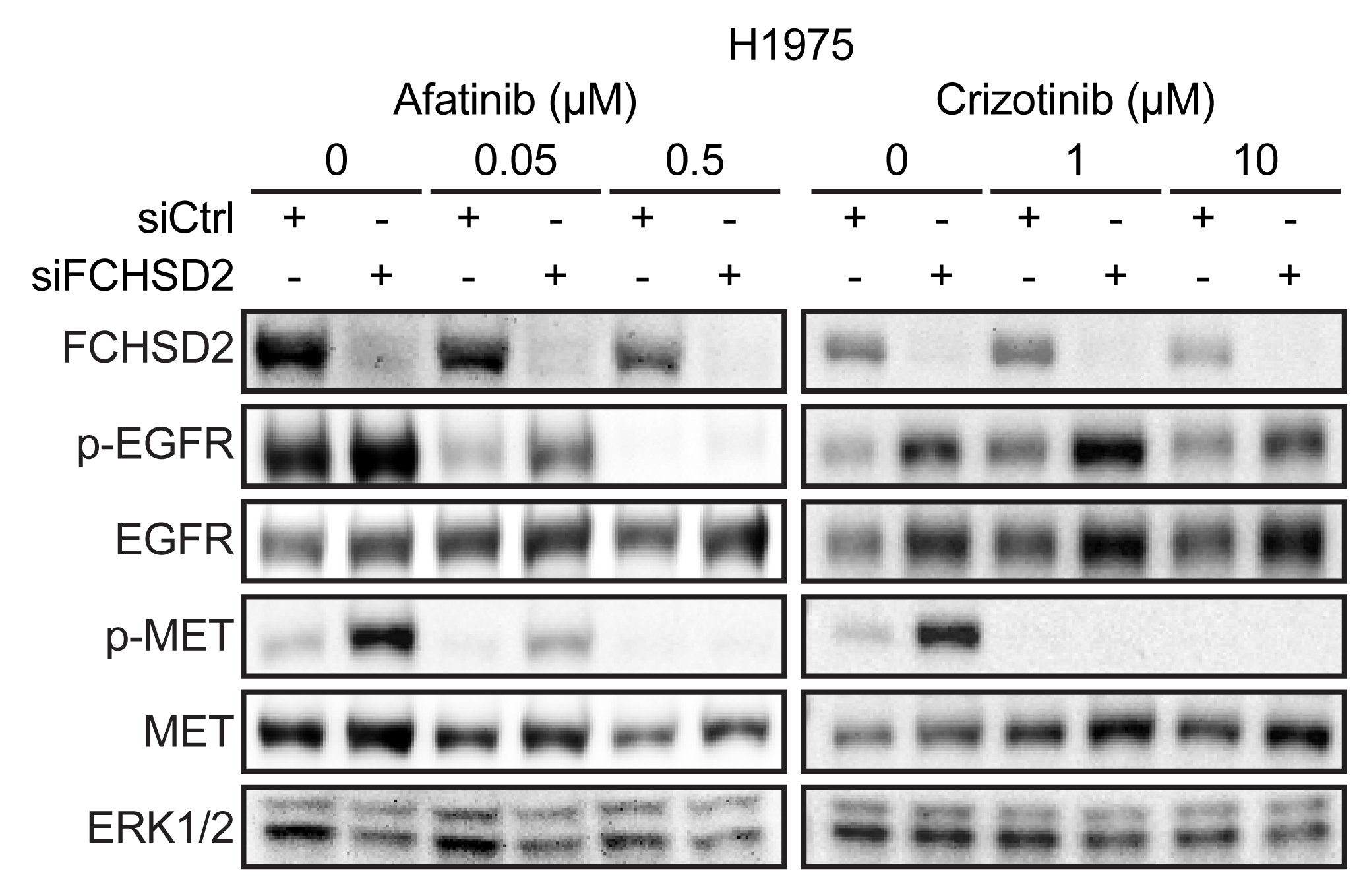

Supplement: S5 Fig — H1975 control or FCHSD2 siRNA-treated cells were incubated with EGFR inhibitor (afatinib) or MET inhibitor (crizotinib) as indicated concentration for 24 h. EGFR, epidermal growth factor receptor; FCHSD2, FCH/F-BAR and Double SH3 Domain-Containing Protein; MET, proto-oncogene c-Met; RTK, receptor tyrosine kinase; siCtrl, control siRNA; siRNA, small interfering RNA. (TIF) [file pbio.3000778.s007.tif]

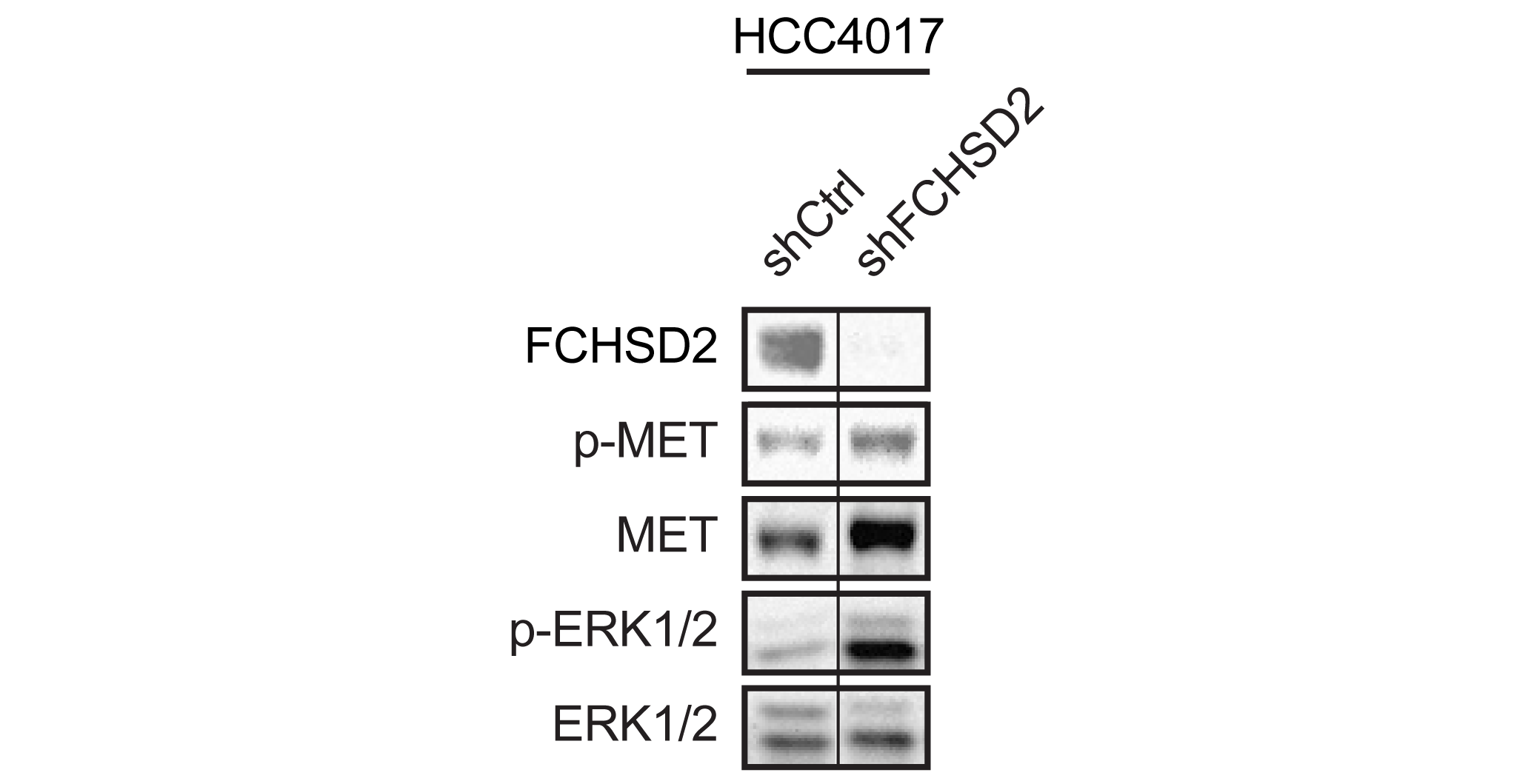

Supplement: S6 Fig — The HCC4017 cells stably expressing shCtrl or shFCHSD2 at steady state. ERK1/2, extracellular signal-regulated kinase 1 and 2; FCHSD2, FCH/F-BAR and Double SH3 Domain-Containing Protein; MET, proto-oncogene c-Met; shCtrl, control shRNA; shRNA, small hairpin RNA. (TIF) [file pbio.3000778.s008.tif]

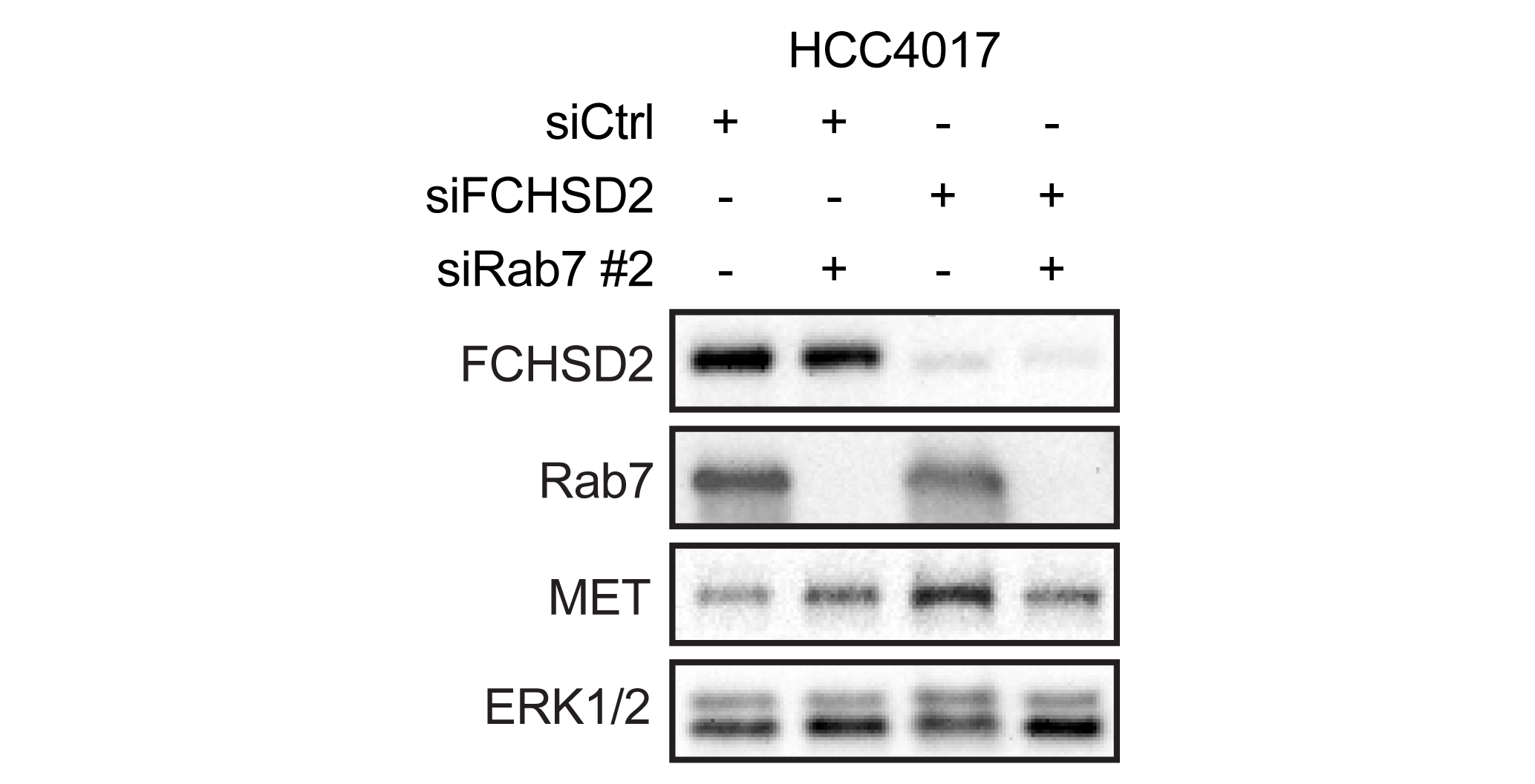

Supplement: S7 Fig — Rab7 KD by a different pool of siRNAs abolishes the MET up-regulation induced by FCHSD2 depletion. FCHSD2, FCH/F-BAR and Double SH3 Domain-Containing Protein; KD, knockdown; MET, proto-oncogene c-Met; Rab7, Ras-related protein Rab-7A; siCtrl, control siRNA; siRNA, small interfering RNA. (TIF) [file pbio.3000778.s009.tif]
