# Supplementary material for: FCHSD2 controls oncogenic ERK1/2 signaling outcome by regulating endocytic trafficking
Source: PLoS Biol. 2020 Jul 17;18(7):e3000778. doi: 10.1371/journal.pbio.3000778 (PMC7390455; doi:10.1371/journal.pbio.3000778)
Supplement: Attachment — Submitted filename: Response to reviewers-final.pdf [file pbio.3000778.s010.pdf]

Responses (in black) to comments (in blue) from reviewers and the editor.

Editor Comments:
Thank you very much for submitting your manuscript "FCHSD2 Controls Oncogenic ERK1/2 Signaling Outcome by Regulating Endocytic Trafficking" for consideration as a Research Article at PLOS Biology. Your manuscript has been evaluated by the PLOS Biology editors, an Academic Editor with relevant expertise, and by three independent reviewers.

As you will see, all the reviewers are very positive and find the conclusions interesting and significant for the field, however they also raise a few points that would need to be addressed with additional experiments to strengthen the results. The reviewers have also requested several clarifications.

In light of the reviews (attached below), we will not be able to accept the current version of the manuscript, but we would welcome re-submission of a revised version that takes into account the reviewers' comments. We cannot make any decision about publication until we have seen the revised manuscript and your response to the reviewers' comments. Your revised manuscript is also likely to be sent for further evaluation by the reviewers.

We expect to receive your revised manuscript within 2 months.

Please email us (plosbiology@plos.org) if you have any questions or concerns, or would like to request an extension. At this stage, your manuscript remains formally under active consideration at our journal; please notify us by email if you do not intend to submit a revision so that we may end consideration of the manuscript at PLOS Biology.

Dear Editor:

We greatly appreciate your handling this manuscript, and especially your willingness to allow us to resubmit despite not being able address all the concerns experimentally, given the pandemic shut-down. Nonetheless we have endeavored to address the concerns of all referees both by changes in the text and by adding more data, which we had on hand. We believe these revisions have improved the manuscript. Our specific responses to each reviewer are detailed below.

Reviewer #1:

Comments:
In the present manuscript, Xiao G. et al., characterized the role of FCHSD2 in endocytosis and trafficking of RTKs. Authors reported that FCHSD2 has a role in ligand-induced trafficking of EGFR and Met receptors, and, in particular, they proposed that it acts as a positive regulator of receptor recycling. They also characterized the role of FCHSD2 in unstimulated basal conditions, where depletion of FCHSD2 causes the increased transcription of EGFR and MET mRNA, possibly through the activation and nuclear translocation of ERK1/2/c-Jun. They also showed that Rab7 is required for the decreased recycling of TfR and EGFR upon siFCHSD2, suggesting that FCHSD2 function could be that of counteracting Rab7. In addition, siRab7 also reverted the increase in EGFR and MET levels induced upon FCHSD2 depletion. FCHSD2 loss correlates

with high grade NSCLC; patients with high FCHSD2 have an elevated survival at variance with patients with high Rab7 that display low survival rates.

The manuscript provides novel insight into the connection between endocytosis and signaling in cancer. Therefore, is of high interest for the cell biology and cancer community. However, there are a number of issues that need to be solved prior publication.

We appreciate the referee's comments that our paper "provides novel insight into the connection between endocytosis and signaling in cancer", that our work "is of high interest for the cell biology and cancer community". We have taken the referee's suggestions for improvement to heart.

1. The assay described in Fig. 1B, C does not measure properly recycling, but it just detects disappearance of internalized Tf/EGF signal. For TfR, which is almost exclusively recycled back to the PM, it could be used as a proxy for recycling. However, this is not the case for EGFR, which is both recycled and degraded after internalization. Thus, the experiment in Fig. 1C suggests that disappearance of internalized EGF signal is delayed, which is compatible with either defective recycling or defective degradation. This methodological issue need to be better discussed. In addition, the nomenclature on the Y axis in Fig. 1B, C need to be changed accordingly. As it is represented now, it is misleading as it seems that recycling decreases with time.

We thank the referee for raising this point regarding EGFR recycling. We have revised the description of this experiment in the result section. *"FCHSD2 KD also reduced the extent of EGFR recycling (Fig 1C, S2A and S2B Fig). However, unlike TfnR, EGFR is both recycled and degraded after internalization [19]. Therefore, to further explore which step(s) along the endocytic pathway were disrupted…"*. We have also changed the nomenclature on the Y axis in all figures, from "TfnR or EGFR recycling" to "Intracellular Tfn or EGF"

2. Fig. 1F and Fig. 2B. It is not clear how the assay is performed. Are all samples (from 0.5h to 6h of EGF stimulation) treated with cycloheximide for the same period of time? As cycloheximide could really affect the biosynthesis of many factors involved in endocytosis, trafficking and degradation (also considering the effects of FCHSD2 on transcription/biosynthesis), I would recommend to repeat EGFR degradation curve (-/+ siFCHSD2) without this treatment.

Cells were treated with cycloheximide for 30 min before EGF stimulation and remained in CHX for the duration of the experiment. This protocol is routinely used to measure EGFR turnover and should not affect the rate of degradation of the pulse of EGFR that is rapidly taken up after EGF addition. Thus, we do not expect to observe differences in the effect of FCHSD2 KD on EGF-induced EGFR degradation measured with or without cycloheximide. We had intended, nonetheless, to repeat these experiment; however, our institution is locked down due to the outbreak of COVID-19.

3. Fig. 3B. The treatment with Afatinib is not convincing, as it is not substantially reducing

EGFR activation (measure as p-EGFR). More consistent inhibition is required to exclude a role for EGFR kinase activity in the process.

We have replaced this gel (new Fig. 3B) with another experiment in which we obtained greater inhibition of p-EGFR at the highest concentration of Afatinib and still detected an increase in EGFR and MET upon siFCHSD2. In 3 separate experiments, we consistently observed increased EGFR and MET even upon inhibition of EGFR activity by Afatinib. Had we not been in lockdown we would repeat this experiment with a second EGFR inhibitor. Nonetheless, we are confident with our finding that EGFR activity is not required.

Also, the WB shown on the left does not reproduce the increase in EGFR and MET upon siFCHSD2 observed in previous experiments.

The new gel shows increased EGFR and MET, which is a highly reproducible observation despite variations in extent and has been quantified in Fig. 3C across several experiments.

As Erk1/2 is involved in the process, another standard loading control should be used.

We agree that another standard loading control would have been better for these experiments. However, although the treatments might affect downstream signaling, including pERK1/2, the total levels of ERK1/2 remain consistent across different conditions, even under ERK1/2 inhibition (see Figure 4A, D and F). Thus, the conclusion should remain unaffected. We now describe this in the Figure legend.

4. Fig. 3D. Authors concluded from this experiment that STAT3 is not involved in the process. However, its phosphorylation is affected upon siFCHSD2. Authors should better discuss their conclusion.

Agreed. We have added the following discussion to our results: According to previous studies, the transfer of active RTKs to perinuclear endosomes can trigger the juxtanuclear activation of a weak STAT3 signal that leads to the required threshold of phosphorylation for nuclear translocation [24, 25]. In turn, accumulation of nuclear p-STAT3 promotes the transcription of HGF and c-Fos [26], leading to upregulation of EGFR [27] and MET [28, 29]. To test whether this signaling pathway accounted for our findings, we treated cells with an EGFR inhibitor (afatinib) or a MET inhibitor (crizotinib). However, neither inhibitor affected the upregulation of RTKs in the FCHSD2 depleted cells (Fig 3B, 3C and S5 Fig). Indeed, FCHSD2 KD decreased the level of p-STAT3 (Fig 3D) and was unable to trigger the transcription of STAT3 target genes, HGF and c-Fos (Fig 3E), perhaps as a result of its rerouting to the LAMP1-positive late endosomes/lysosomes at the expense of its accumulation in perinuclear Rab11-positive recycling endosomes (Fig. 2A).

5. Fig. S1. The inhibition of Erk1/2 with SCH compound has the same effect of the Rab7 inhibition on TfR recycling (in Fig. 5A). It seems that the decrease in TfR recycling upon siFCHSD2 requires the action of Erk1/2, as it is reverted by SCH treatment. I did not understand how the authors interpret this results in Fig. S1 at variance with results shown in Fig 5 A.

The referee correctly describes our findings. Our interpretation is that in the absence of FCHSD2, ERK inhibition has the same effect on recycling as in its presence (compare the differences between siCtrl +/-SCH and siFCHSD2 +/-SCH). Therefore, we hypothesize that the effects of ERK1/2 on TfnR recycling are through other downstream effectors, instead of FCHSD2.While we prefer not to speculate in the text, a possible candidate for this effect, based on the literature is GGA3 (Golgi-localized, Gamma-ear-containing, Arf-binding protein 3), which has been shown to be regulated by ERK1/2 activity and may affect the recycling pathway (Kametaka *et al*., *Mol. Cell Biol.*, 2005, PMID: 16135791). Additionally, in preliminary results (n=2), we found that the TfnR recycling remained unaffected in the cells overexpressing the phosphorylation mutants of FCHSD2 (FCHSD2-S681A and S681E, see Xiao *et al*., *PNAS*, 2018, PMID: 30249660). A

In addition, in Fig. S1, the effects of SCH treatment are mild - if any - as well as the effects of siFCHSD2 in ARPE-19 cells. The magnitude is minimal and, although some time points are statistically significant, it is hard to draw conclusion on the biological significance of these effects. In my opinion, these data on SCH treatment should be more critically discussed.

We agree the effects are smaller, but still significant. We were mainly focused on the differences (Control siRNA +/- SCH; siFCHSD2 +/- SCH) which show that SCH significantly increases the rate of TfnR recycling, and indeed to the same extent, under both conditions in ARPE-19 cells. We expanded the description of this experiment to a full paragraph in the text for greater clarity and accuracy.

6. Fig. 4F. The WB provided is not completely convincing. The levels are variable and there are some inconsistencies. For instance, MET levels seem not to be rescued by both treatments in H1975, just all levels are decreased. Similarly, in HCC4079, MET levels are variable, sometimes increasing (SCH) sometimes decreasing (GSK) after treatments. It is not clear what these treatments are really doing. In addition, the blots are not representative of the quantification on the right. In the quantitation, samples should be all normalized to the same reference, i.e. the not treated Ctrl sample. An important experiment that would corroborate this finding is the analysis of EGFR and MET mRNA levels upon siFCHSD2, to investigate if they are rescued by these treatments.

We observed some variations from different batches of cells in these experiments, although we tried our best to keep all the conditions consistent. Therefore, the experiments were repeated multiple times to confirm the observations and quantified in Fig. 4G. We also observed differences between cell type responses that may result from a great deal of heterogeneity among different NSCLC lines with regard to signaling. We note that the corresponding increase in the loading control for HCC4017 cells treated with SCH, and the apparent increase in MET upon SCH treatment is not significant or reproducible. We have, nonetheless, re-analyzed the data as the referee suggested. We agree with the referee's suggestion that incorporating the mRNA level data to this study would greatly strengthen the findings. However, our institution is locked down due to the outbreak of COVID-19. Unfortunately, we could not perform any experiments to test that in this difficult time.

7. The effect of siRab7 are strong and convincing. However, it is not clear how Rab7 function is linked to Erk1/2 nuclear translocation and activation, and to the induced EGFR/MET transcription. The model shown in Fig. 6A is too complicated and does not convey the message. Which is the mechanism though which Rab7 KD revert the increased EGFR and MET levels? Authors should show if siRab7 is able to revert the MET and EGFR mRNA levels. Does siRab7 revert the increased Erk1/2, c-Jun, Ets-1 levels in the nucleus?
These connections need to be investigated and further discussed in the text.

We have revised the model in Fig. 6A. We simplified the model and highlighted our main findings in this study. We agree with the referee's suggestion that we should test if Rab7 KD reverts the increased EGFR and MET mRNA levels and reverts the increased nuclear ERK1/2, c-Jun, Ets-1 levels in FCHSD2 KD cells. Unfortunately, we could not run any assays to test that in this difficult time. We have indicated the value of these additional experiment in the Discussion. As to the mechanism by which FCHSD2 regulates Rab7, this is the subject for future experiments. We have indicated this in the Discussion, but refrained from unwarranted speculation.

Reviewer #2:

Comments:
This is an excellent study interrogating how membrane trafficking alterations downstream of FCHSD2 depletion impact ERK1/2 signaling. The primary conclusions are that FCHSD2, by negative regulation of Rab7, diverts tyrosine kinase receptors (TKRs) from late endosomes, limiting access of TKRs to ERK1/2 activation at the late endosome. Loss of FCHSD2 increases traffic of TKRs to LE and subsequent activation of ERK1/2. The data, for the most part, support the stated conclusions. My main comments are as follows.

We thank the referee for carefully reading the manuscript as exemplified by the above succinct and accurate summary. We also appreciate that the referee considers our data to be consistent with our stated conclusions.

1. The authors should be more attentive to alerting the reader what was done in the KRAS/p53 line and what was done in EGFR mutant. For the non-cancer specialist, the potential importance of the distinction might be lost. The authors might also consider adding a discussion paragraph on the topic of the oncogenic driver mutation and the impact of FCHSD2 (loss thereof).

These cell lines were chosen as they have upregulated MAPK signaling, which we thought would be important for FCHSD2 activity. We have more clearly stated our rationale in choosing these two cell lines in the Introduction and Discussion. We have also indicated in the main text which cells we're using for each experiment. The results obtained with both are consistent; however, the effects are often stronger in the Kras mutant expressing HCC4017 cells, which is consistent with our hypothesis of increased ERK activation downstream of Kras in late endosomes. We have discussed these differences in the Discussion and indicated that further experiments would be needed to determine whether and to what extent our findings can be extended to other NSCLC lines and to other cancers.

2. The authors might consider extending the main conclusion, that KD of FCHSD2 increases expression of EGFR and MET, in analyses of other human NSCLC cell lines, both EGFR mutant and KRAS.

As stated above, it would be great to extend our analyses to other NSCLC cell lines. However, our institution is locked down due to the outbreak of COVID-19. Unfortunately, we could not perform any experiments for that in this difficult time. We have added this suggestion for future experiments in our discussion.

3. The authors might provide some speculation (or perhaps data) on how FCHDS2 acts as a negative regulator of Rab7. Does FCHSD2 activate a Rab7 GAP or inhibit a Rab7 GEF?

We thank the referee for raising this point. As we have no specific information we hesitate to speculate, but we have added a paragraph regarding general mechanisms by which this might happen (direct, indirect inhibition of GEFs or activation of GAPs). These are worthwhile experiments for the future.

4. Fig 6 is not particularly strong, and I do not feel it strengthens the paper but rather detracts from the otherwise strong cell biology. To appreciate the data in panel B one would want to see expression of FCHSD2 in adjacent normal lung tissue. Pushing a link between the expression of a single protein (FCHSD2) and grade is not particularly compelling. I assume the analyses in Fig6C are based on RNAseq. Have they correlated IHC with RNAseq in their own cohort (panel B) to establish the RNAseq data agrees with the IHC? The author's need to present data on the expression distribution of FCHSD2 and Rab7 so the reader can assess how meaningful the distinction is. Did the author's use above and below the median or upper and lower quartiles? They should also look at the TCGA database to determine how specific this is for lung.

We appreciate the referee's comment, but we think these experiments help to link our in vitro mechanistic studies to human cancers. However, we have commented on the limitations and been careful to indicate that these are correlative studies. We note that neither of the other two referees expressed concern about this data.

5. Throughout the manuscript the authors should reanalyze the recycling curves. In many instances it looks like the extent of recycling rather than the rate of recycling is changed. For example, in Fig 1B it is the extent of transferrin receptor recycling that is changed by FCHSD2 depletion not the rate, suggesting a fraction of transferrin receptor is trapped and no longer recycled rather than a slowing of recycling. Although I do not think it will change the conclusions it might change how one thinks about the molecular mechanism(s) underlying the effects.

We agree with the referee's point that in many cases FCHSD2 KD mainly affected the extent of receptor recycling rather than the rate of recycling (the kinetic curve looks similar between siCtrl and siFCHSD2 cells). These results are consistent with our observations in immunofluorescent data that a fraction of EGFR or MET is trapped in endosomal compartments in FCHSD2 KD cells. We have carefully revised our interpretation about these finding in the text.

Reviewer #3:

Comments:
This is a clear, concise, well-executed study, exactly what we have come to expect from Dr. Schmid and her colleagues. I was left a little wanting, how exactly does the FCHSD2-depeletion induced upregulation of the RTKs occur, what specifically does Rab7 do in this pathway, why the seeming conundrum between increased trafficking to late endosome/lysosome and yet upregulation of expression? But there is valuable information in this study and no one study ever has all the answers

We sincerely appreciate the positive comments from the referee.

1. Abstract. "…can reciprocally regulate endocytic trafficking by creating feedback loops in non-small-cell lung cancer (NSCLC) cells…". The sentence is awkward. Presumably these feedback loops are present in other cancer cells, perhaps in non-cancer cells.

Agreed. We have revised the sentence in the abstract for clarity.

2. Line 53, can you please be more specific as to what you mean by "…cancer-specific activation…", in particular, what is meant by activation?

We previously showed that phosphorylation of FCHSD2 on S681 by ERK1/2 was required for its function in clathrin-mediated endocytosis and that this was true only in the cancer cell lines we examined, but not in nontransformed APRE cells. We have clarified this in the text.

3. Figure 1 and throughout, use 2 siRNAs or better describe the specificity of the single one used or perhaps validate 1-2 key phenotypes in a knockout line.

We also observed increased MET expression and enhanced ERK1/2 activity at steady-state in the HCC4017 cells stably expressing shFCHSD2 compared to the cells with shCtrl expression. We have included these data in new S6 Fig., related to Fig. 4A.

4. Figure 1D, please, a word or two about the specificity of the PO4-EGFR antibody in the results section.

We have added the description about the specificity of the antibody to the results. *"...we utilized an antibody specifically targeting phosphorylated EGFR at tyrosine 1068 to follow p-EGFR trafficking using immunofluorescence."*

5. The upregulation of mRNA in Figure 3 is relatively minor, and in light of the enhanced degradation its seems the balance should not be towards such a strong upregulation of the protein. Please discuss.

EGFR and MET degradation were measured in the presence of EGF and HGF to induce their rapid internalization. mRNA levels were measured at steady state. In Figure 3A, we observed an

~1.5-fold increase in EGFR mRNA and > 2-fold increase in MET mRNA in the FCHSD2 KD cells compared to siCtrl cells at steady-state. In Figure 3C, we normalized the protein expression of EGFR and MET and observed a similar fold change in FCHSD2 KD cells compared to siCtrl cells at steady-state. We believe that the correlation between mRNA and protein levels is reasonable.

6. Figure 5, one Rab7 siRNA?

We used another pool of Rab7 siRNA from different provider (Sigma) and observed the similar results. We have included these data to S7 Fig., related to Fig. 5B and described this in the main text.

7. Figure 5, higher levels of active Rab7 in FCHSD2 knockdown cells. It would be nice if there was some additional data here. What explains the activation, regulation of a GEF or a GAP?

Referee #2 also raised this point. We believe deciphering this mechanism is beyond the scope of this paper, but have included a new paragraph to discuss the potential general mechanisms in the text. We have refrained from making any more specific speculations in the absence of any data.

8. Figure 5. There is some uncertainty regarding the specificity of the antibodies recognizing the active Rabs. The results state that an antibody specific to active Rab7 was used without reference or validation. The authors need to address this issue.

Here we used the commercial Rab7 Activation Assay Kit (#NEBB40025, NewEast Biosciences). The manufacture provides the data to demonstrate that the anti-active Rab7 monoclonal antibody could only immunoprecipitate the purified GST-tagged Rab7 proteins after treated with GTPγS but not the Rab7 proteins after treated with GDP. While we had planned to confirm this ourselves, our institution is locked down due to the outbreak of COVID-19. Unfortunately, we could not perform any experiments to test that in this difficult time. According to the validation by the manufacture, we think the antibody should be fine.
