# Supplementary material for: FCHSD2 controls oncogenic ERK1/2 signaling outcome by regulating endocytic trafficking
Source: PLoS Biol. 2020 Jul 17;18(7):e3000778. doi: 10.1371/journal.pbio.3000778 (PMC7390455; doi:10.1371/journal.pbio.3000778)
Supplement: Attachment — Submitted filename: Response to Production Staff.docx [file pbio.3000778.s011.docx]

We have addressed all of the production requirements, as described below.

1. Please put all reference citations in square brackets, with no spaces, separated by either commas or dashes, e.g. [1,2,3] or [1-3].

Done.

2. Please ensure that all supporting information legend titles use the following format verbatim in all in-text citations (including spelling and capitalization): S1 Fig, S1 Table, S1 Text, S1 Data, S1A Fig etc. Each file type should be numbered starting at 1.

Done

3. Please note that your submission cannot contain any references to generic "supporting information" files and instead must refer to specific files using our naming convention (S1 Fig, S1 Table, S1 Text, S1 Data, etc.)

Done

4. Place each main figure legend directly below the paragraph in which that figure is first referenced.

 	Done
